# Legal, Regulatory, and Practical Issues to Consider When Adopting Decentralized Clinical Trials: Recommendations From the Clinical Trials Transformation Initiative

**DOI:** 10.1007/s43441-019-00006-4

**Published:** 2019-12-09

**Authors:** Maria Apostolaros, David Babaian, Amy Corneli, Annemarie Forrest, Gerrit Hamre, Jan Hewett, Laura Podolsky, Vaishali Popat, Penny Randall

**Affiliations:** 1 Pharmaceutical Research and Manufacturers of America (PhRMA), 950 F Street NW, Suite 300, Washington, DC 20004 USA; 2 Advarra Consulting, 1501 Fourth Ave, Suite 800, Seattle, WA 98101 USA; 3 Clinical Trials Transformation Initiative, 200 Morris St, Durham, NC 27701 USA; 4 Department of Population Health Sciences, Duke Clinical Research Institute, 215 Morris St, Suite 210, Durham, NC 27701 USA; 5 Hamre Strategies LLC, 507 N Elizabeth St, Durham, NC 27701 USA; 6 Office of Compliance, Center for Drug Evaluation and Research, Food and Drug Administration, New Hampshire Ave, Building 51, Silver Spring, MD 20903 USA; 7 Science 37, Inc., 12121 Bluff Creek Dr, Suite 100, Los Angeles, CA 90094 USA; 8 Center for Drug Evaluation and Research, Office of New Drugs, Food and Drug Administration, New Hampshire Ave, Building 22, Silver Spring, MD 20903 USA; 9 IQVIA, 10188 Telesis Court, Suite 400, San Diego, CA 92121 USA

**Keywords:** Mobile clinical trials, Mobile technology, Mobile medical applications, Mobile nursing, Participant-centric trials, Telehealth

## Abstract

**Background:**

Traditional clinical trials are often expensive, inefficient, include selected populations, and can create significant participant burden via travel and other logistical demands. Using new technologies and methodologies to promote a decentralized approach has the potential to improve the efficiency of clinical trials. The Clinical Trials Transformation Initiative (CTTI)—a public–private partnership to improve clinical trials—launched a multi-stakeholder Decentralized Clinical Trials (DCTs) Project to provide recommendations on addressing the actual and perceived legal, regulatory, and practical challenges with DCT design and conduct in the United States.

**Methods:**

Informed by qualitative group interviews and an expert meeting, CTTI engaged stakeholders to identify key challenges to implementing DCTs and possible solutions.

**Results:**

The CTTI DCT project team used the interview findings and expert feedback to develop recommendations that will drive broader use of DCTs.

**Conclusions:**

CTTI’s recommendations cover protocol design, use of telemedicine and mobile healthcare providers, medical product supply chain, investigator delegation and oversight, and safety monitoring considerations. By implementing these recommendations, sponsors, contract research organizations, and others can help advance successful medical product development using mobile technologies and methodologies in DCTs.

## Introduction

Remote or decentralized clinical trials (DCTs) have gained attention as technology, infrastructure, and knowledge have developed to support their use. DCTs—defined as trials executed through telemedicine, mobile/local healthcare providers (HCPs) and/or mobile technologies—are not bound by the geographic limitations that affect traditional trials. Therefore, they can recruit participants from anywhere, potentially resulting in accelerated enrollment and more diverse participants' representative of the target population. Moreover, measurements can be more frequent or even continuous because they are not restricted by scheduled clinic visits.

A decentralized approach allows trial participants to take part in clinical research from anywhere, with research activities better integrated into their daily routine. DCT approaches may lessen participant burden (e.g., travel costs and time loss), which may enhance retention and facilitate certain research that may otherwise be unduly burdensome under traditional clinical trial constructs [1].

Despite the potential benefits of DCTs, adoption has been slow and variable. Some barriers may be immature digital infrastructure, limited experience with the approach, and the perception of regulatory barriers with implementing and using data from DCTs.

Importantly, almost all states now have telemedicine laws that allow for mobile medicine, which sponsors, CROs, and other stakeholders can use to inform DCTs [2, 3]. However, effort is needed to drive acceptance and implementation of DCTs.

The Clinical Trials Transformation Initiative (CTTI; www.ctti-clinicaltrials.org), a public–private partnership co-founded by Duke University and the United States (US) Food and Drug Administration (FDA), whose mission is to develop and drive adoption of practices that will increase the quality and efficiency of clinical trials, recognized an opportunity to encourage broader use of DCTs. CTTI launched the Decentralized Clinical Trials project, guided by the following objectives: (1) identify perceived and actual legal, regulatory, and practical barriers to conducting DCTs and (2) identify opportunities to clarify and inform policies that affect the implementation of DCTs. This project is one of the several projects [4] developed by CTTI to address challenges with planning and conducting clinical trials using mobile technologies.

## Materials and Methods

### Industry Interviews

Between October 25, 2016, and January 20, 2017, CTTI conducted semi-structured group interviews with trial sponsors to identify important legal and regulatory challenges they faced at that time in conducting DCTs, and their potential solutions. CTTI chose to conduct group interviews with multiple individuals from the same company to enable a multi-faceted discussion during a single interview. A total of 31 purposefully selected [5] representatives from seven different pharmaceutical or biotechnology companies that were currently implementing or planning DCTs participated in nine group interviews (2–7 representatives per group). The Duke University Health System Institutional Review Board (IRB) designated the interviews “exempt” from full board review.

For those companies that had experience in the use of some remote clinical research components, representatives described barriers faced and lessons learned. For those companies that had less experience with remote components, representatives described the types of considerations their companies have discussed in preparation for conducting such research in the future and barriers faced. All interviews were audio recorded and transcribed verbatim. For some topics, a simple summary was produced describing representatives’ experiences. For other topics, data were analyzed using applied thematic analysis [6]. Challenges and suggested solutions that emerged from the interviews are described in Table 1. Most of the challenges identified by representatives currently are still factors in the planning and conduct of DCTs; a few, however, no longer represent considerable challenges (see sections on *Engage and Partner* and on *Telemedicine State Licensing*).

**Table 1.** Challenges and Suggested Solutions from the Interviews and Considerations from the Expert Meeting.

Interviews: Main Perceived Challenges
• Ensuring that protocol-defined activities are carried out in a consistent manner throughout the study when relying on mobile HCPs, given the potential for varying medical qualifications of these providers and/or inconsistencies in their knowledge of the protocol • Remotely replicating the interactive part of the informed consent process, allowing investigators to gauge participant understanding and ensure that participants are adequately informed • Verifying trial participants’ identities and ensuring their privacy and confidentiality when research is completely remote • Identifying how to monitor safety within the context of remote clinical research • Planning for and implementing clinical research with telemedicine components may be difficult and time consuming due to inconsistent state telemedicine laws • Some states require a “supervising” physician be licensed to practice medicine in their state • Some states do not allow direct shipment of IMP to trial participants • Within states that allow the direct shipping of IMP to trial participants, IMP receipt and accountability is difficult because study sites are not involved in tracking the details of when an IMP is received by a study participant
Interviews: Main Suggested Solutions
• Starting trial planning early: • Engaging partners, collaborators, and stakeholders (including legal and regulatory) at the earliest stage of the clinical research trial planning and design • Reviewing and understanding individual state laws governing clinical trials, medical practice, distribution of IMP, and telemedicine • Developing systems for tracking receipt and drug accountability in remote trials • Enhancing current systems to include training and assessments for mobile HCPs • Adjusting current systems to include remote safety monitoring and privacy and confidentiality procedures • Using a problem-based design approach. For example, start with the design and build from that, rather than trying to add devices into an already established protocol • Using a participant-centered approach. For example, obtain participant feedback throughout trial design and implementation • Delineating the delegation of investigator responsibilities in the context of remote clinical research • Identifying physicians with medical licenses in multiple states • Clarifying federal regulations and standardizing state laws: • Clarifying guidance on the distribution, shipping, disposition, etc. of IMP within the context of remote clinical research • Allowing for more reciprocity between states • Staying focused and keeping the plan simple.
Considerations from the Expert Meeting
• Engage trial participants and regulatory agencies early in the trial design and development phase • Develop consensus on definitions for terms that are central to DCT design and conduct, e.g., what defines an “investigational site” in a DCT? • Glean inspiration and lessons learned from current and previous successful DCTs for implementing new DCTs • Consider fit-for-purpose study designs or starting with a DCT in which the safety profile of the IMP is well known • Highly varied state laws and regulations need to be thoroughly understood and recorded in an accessible location, e.g., a public database • Tasks or activities provided by third-party vendors may be leveraged when thoughtfully integrated in DCT design • Trials with a mobile component should be held to the same standards as traditional trials • Guidance from regulatory bodies is needed to define investigator responsibilities regarding participant care oversight and potential delegation of activities

*DCT* decentralized clinical trial, *HCP* healthcare provider, *IMP* investigational medical products.

### Expert Meeting

CTTI convened an expert meeting in July 2017 to discuss the interview findings and experience of those practiced in conducting DCTs. The 50 participants represented a variety of stakeholders. Meeting participants possessed knowledge of or experience with the perceived and actual legal and regulatory challenges associated with designing or conducting DCTs. Key themes from the discussion included those in Table 1. Meeting participants discussed evidence-based solutions to inform the development of project recommendations and resources to address legal and regulatory challenges currently associated with DCTs [7].

## Results

### Recommendations

Based on key topics and themes that emerged from the qualitative interviews and multi-stakeholder expert meeting [8], the CTTI developed consensus recommendations [9] around 6 DCT topics (Table 2):

**Table 2.** CTTI Recommendations and Considerations for Decentralized Clinical Trials.

Approaches and Protocol Design	The design and implementation of DCTs does not have to be an all-or-nothing approach. Use a partially decentralized (hybrid) approach if applicable Engage all stakeholders early and often Implement fit-for-purpose designs (see also Table 3) Proactively address and map data flow and communications Partner with those experienced with telemedicine
Telemedicine State Licensing	Maintain an investigator in each state in which the DCT is conducted Utilize investigators licensed in multiple states Contract with qualified mobile HCP research services Consult appropriate experts regarding telemedicine laws Seek reliable legal expertise and/or partnerships
Direct-to-Trial Participant IMP Accountability	Consult and ensure compliance with relevant federal and state statutes and regulations Clearly describe IMP procedures in the protocol Outline accountable parties at each step of the supply chain in the Investigational Plan Engage vendors/pharmacies with direct-to-trial participant experience
Mobile Healthcare Providers	Consider as a substitute for visits to investigative sites Delegate responsibilities consistent with state laws and the protocol, and only to qualified personnel Consider consulting/partnering with a mobile HCP vendor
Investigator Delegation and Oversight	Hold to the same standards as traditional trials Define “routine care”/“practice of medicine” as opposed to “clinical trial-related activities” clearly in the protocol Evaluate local and/or mobile HCP’s role in clinical trial and in relationship to FDA regulations Delegate authority and responsibilities in the same way as for traditional trials Consult FDA regulations and guidance when determining whether or not and how to list HCPs on the Form FDA 1572
Safety Monitoring	Hold to the same standard as traditional trials Clearly articulate remote safety monitoring procedures and train investigative staff Establish record-keeping protocol to ensure compliance Develop protocol-specific safety monitoring and communication escalation plans

*DCT* decentralized clinical trial, *FDA* Food and Drug Administration, *HCP* healthcare provider.

1. DCT approaches and trial design
2. Telemedicine state licensing issues
3. Drug supply chain
4. Mobile HCPs
5. Investigator delegation and oversight
6. Safety monitoring.

### DCT Approaches and Protocol Design

#### Partially Decentralized/Hybrid Approaches

The design and implementation of DCTs need not be an “all-or-nothing” approach. A fully decentralized approach may not include a central physical trial site, but include trial visits conducted via telemedicine or by mobile or local HCPs and the use of mobile technologies to record data. Partially decentralized or hybrid approaches combine some of the above-mentioned features with more traditional approaches. These hybrid approaches may include the following:

- a designated trial site at which specific trial-related activities occur (e.g., chest X-rays) while allowing other procedures (e.g., blood draws, treatment administration) to be completed elsewhere,
- data collection both within and outside of the clinical setting using mobile technologies, and/or
- trial participants and investigative site personnel interacting both at a clinical site and via video or teleconferencing.

Such hybrid approaches can increase trial flexibility.

A DCT will also require some fit-for-purpose protocol design and conduct considerations (Table 3). Consider incorporating DCT features within a traditional trial by introducing remote methodologies with an amendment to an existing protocol where infrastructure is already established, and the safety is well characterized. This will allow investigators/sponsors to gain logistics experience, evaluate user compliance, and compare quality of data to data from traditional methodologies. Sponsors and trial designers can consult use case examples [10–13] and recommendations for best practices for selecting the appropriate technology [14] for a trial. Importantly, patients and sites should be actively engaged in planning for the scientific and operational design and conduct of decentralized trials from the earliest stages of planning a clinical trial using mobile technologies. Additional details are available in CTTI’s recommendations on Optimizing Mobile Clinical Trials by Engaging Patients and Sites (www.ctti-clinicaltrials.org).

**Table 3.** Considerations for Designing a Decentralized Clinical Trial.

1. Determine which activities must occur at the investigative site, which can be performed by a local or mobile HCP, and which are amenable to mobile technology solutions 2. Implement additional trial safeguards, processes, training, and/or procedures to ensure that the protocol is conducted in a compliant manner 3. Assign accountability for the management of source documents at decentralized sites 4. Designate where and how local source documents and electronic information will be stored 5. Plan for needed technological support including training and troubleshooting for all parties and ensuring data integrity with device use and use of electronic systems 6. Consider regional differences in telecommunication availability	

*HCP* healthcare provider.

#### Data Reliability, Integrity, and Traceability

Data reliability and integrity can be a concern in DCTs, which is why it is especially important to proactively address and map data flow, user access controls, data reconciliation, and storage [15]. Sponsors, CROs, and other parties (e.g., information technology vendors) handling data should control and manage data flow (e.g., data use agreements, service level agreements), as data in DCTs may be transferred to and stored among several different parties, locations, and systems. It may be prudent to start with the trial source data and then map data flow, reconciliation, and storage based on how data reliability and integrity are assured, including data control and security (see CTTI’s Mobile Technologies recommendations [14]). This information will also be of interest to IRBs and should, if practicable, be communicated in general terms within the informed consent. DCT operators must also maintain compliance with data privacy and security regulations (e.g., Health Insurance Portability and Accountability Act [HIPAA]). As these are continuously evolving, particularly at the state level, those operating DCTs must ensure they maintain up-to-date knowledge.

#### Engage and Partner

When considering the implementation of decentralized components to optimize clinical trial design, it is important to engage with all stakeholders early in the protocol design process, including meeting with regulatory bodies [16–21], understanding prospective participant perspectives [22], and engaging with experienced vendors. Partnering with investigational sites that are familiar with telemedicine can help optimize implementation. Additionally, as telemedicine is well-utilized in several therapeutic areas (e.g., dermatology, psychiatry, stroke management) [23–32], insight may be gleaned from the standards and practices developed in those fields. It may also be valuable to engage telemedicine providers in protocol development.

### Telemedicine State Licensing

DCTs operating across multiple US states necessitate management of state-by-state licensure requirements for participating practitioners. HCPs, including investigators and their delegates, must be licensed in the state in which they provide trial-related medical intervention to participants. An investigator cannot deliver investigational medical products (IMP) or prescribe treatment to a trial participant in a state in which the investigator is not licensed. To manage state-by-state medical licensure requirements, DCTs that operate across multiple states can maintain an investigator in each state where services are anticipated, utilize investigators licensed in multiple states, use the pathway provided by the Interstate Medical Licensure Compact to expedite licensure for investigators in multiple states [23], and/or contract with companies providing licensed mobile HCP research services in states where the trial will be conducted. With these considerations, investigators must, in fully meeting their trial-related responsibilities, also maintain compliance with applicable laws, regulations, and local standards of practice.

Sponsors planning to incorporate telemedicine in clinical research should be informed of the landscape of applicable laws. In recent years, telemedicine laws have become increasingly uniform across the states: most states permit the use of telemedicine provided that, in doing so, the HCP can meet the standard of care. Nonetheless, important differences remain across states with respect to telemedicine and by extension, DCTs. The selection of states in which to conduct a DCT is a critical strategic decision that will in part be influenced by legal considerations as well as the ability to reach the specific participant population of interest. A primary difference across states is whether the state requires that the provider–participant (or investigator–participant) relationship be initiated in person before shifting to telemedicine, versus allowing that relationship to be initiated through a telemedicine visit. This distinction has implications for DCT design. Sponsors are encouraged to review the laws of each state in which they intend to operate the trial to ensure compliance with applicable laws. Sponsors should consider using online resources of policy organizations that specialize in telemedicine laws [3, 33]. Reliable legal expertise is also recommended to track changes in these laws. This expertise may be obtained from external legal consultants and/or companies that track and report state-by-state changes in laws and regulations.

### Investigational Medical Product Accountability

For DCTs involving IMP delivery directly to trial participants, additional challenges regarding IMP accountability may need to be addressed. IMP accountability and dispensing laws and regulations vary depending on state statutes, and regulations differ according to the product’s registration status with the FDA (investigational or approved) or legal status in a particular state. CTTI recommends reviewing state law requirements for direct-to-trial participant shipping, developing processes to ensure compliance with existing regulations, and engaging with appropriate regulatory bodies early in trial planning and design (e.g., requesting Type B meetings for pre-investigational new drug applications (pre-IND) with the FDA).

The feasibility of IMP delivery directly to the trial participants may also depend on practical considerations, including the product’s nature and stability (e.g., a stable, ready-to-use compound versus one requiring fresh constitution) as well as protocol design. Furthermore, some IMPs are not amenable to direct-to-participant shipment because of route of administration (e.g., IV) or significant or unknown safety profile.

Pathway planning and documentation are critical. Procedures for direct-to-trial participant IMP shipment should be described in the protocol so that the process is clear to the investigator, IRB, and applicable regulatory agencies. Similar to traditional trials, formal standard operating procedures (SOPs) tied to the clinical trial protocol should also be utilized to outline accountable parties at each step of the supply chain, from the administration order through distribution to the participant and recovery of the IMP or container. Different SOPs may be necessary for different DCT scenarios; however, the SOPs should always comply with applicable federal and state regulations.

Organizations may choose to engage a management vendor with experience in direct-to-trial participant shipment. This vendor should have pharmacy licenses in all US states where their services will be utilized. When this is not feasible, sponsors/CROs should engage a central pharmacy through which shipments can be made directly to trial participants.

Whether IMP distribution is handled “in house” or by a third party, sponsors should consider how those responsible will interact with the participant. Relevant considerations include how to handle the IMP once received by the participant (e.g., ensuring that the IMP is intact and stored appropriately), what participants should do with unused IMP, and who participants can contact if there are problems or questions with the IMP (e.g., the package is damaged in transit). Patient-centered interactions are important because they may impact the quality, reliability, or integrity of the data. For example, if the investigator–participant telemedicine interactions are convenient, comfortable, and otherwise positive for the participant, but the participant’s experience with the IMP is burdensome and difficult, the DCT may fall short of its potential to improve retention, compliance, and the overall participant experience.

### Mobile Healthcare Providers

Visits from mobile HCPs may be an appropriate substitute for selected clinical trial visits to investigative sites and may promote participants’ compliance and retention by providing convenience and comfort in the home, office, or in certain circumstances while traveling out of town. As with traditional trials, the investigator is responsible for ensuring that trial procedures are conducted consistently according to the investigational plan. Activities that mobile HCPs may be able to perform include clinical assessments, blood draws, IMP or treatment administration, participant education, and in-home compliance checks. Tasks should be delegated to qualified personnel required by the protocol, as informed by scope of practice parameters defined by applicable state law. Mobile HCPs should be trained on good clinical practice, trial-specific requirements, human participant protections, data protection, and clinical trial billing. Trial operators utilizing mobile HCPs should develop SOPs focused on applicable activities, such as specimen storage and shipping by such providers, as well as basic policies around travel and accommodations. To use mobile HCPs effectively, sponsors and trial operators should consider consulting or partnering with a mobile HCP vendor with experience in clinical trials. Mobile HCPs may offer a way for prospective trial participants to participate in trials regardless of trial duration; frequency of visits; disease state; distance to travel to the investigative site; school, work, or family obligations; or vacation/travel plans.

It should be noted that concerns regarding oversight and liability can complicate the use of mobile HCPs. For simplicity, sponsors should consider engaging PIs that have existing capability within their practice to integrate mobile care into the trial (whether locally or, less likely, over a broad geographic area), without need to involve a third-party vendor. Otherwise, sites may be hesitant to act as the IRB of record for a sponsor-engaged vendor over which it has little actual authority, even if the sponsor offers contractual indemnification related to that activity, and instead try to disclaim potential liability caused by decentralization. The vendor should prospectively identify who will provide the service (e.g., who will act as a research team member) to mitigate concerns. The site’s clinical investigator may have other concerns about meeting requirements for ensuring adequately qualified staff, providing training on the protocol, and assuring the integrity of study data. Where a local IRB declines to assume oversight responsibility for such study activity, the sponsor may need to engage a central/independent IRB to review the home health portion of a protocol to be performed by mobile HCPs, and likewise, where the local PI declines to assume oversight responsibility for such study activity, a PI affiliated with the mobile HCP may need to be designated.

### Investigator Delegation and Oversight

DCTs using telemedicine or mobile HCPs should not be held to a different standard than in traditional trials with regard to investigator delegation and oversight. As with traditional trials, standard considerations exist prior to determining delegation, including the IMP development phase, clinical complexity and vulnerability of the study population, safety profile of the IMP, and trial endpoints. Additionally, consideration should be given to the capabilities of those to whom authority and responsibility is delegated to implement DCT methodologies reliably and effectively.

For DCTs, additional considerations may be required to ensure that adequate resources are available for investigative sites potentially enrolling increased participant populations. To protect participants’ rights, safety, and welfare, IRBs will be particularly interested in probing whether the investigative sites have the capacity in human and technological capital to implement the trial as designed, address adverse events, and ensure the conduct appropriately minimizes risks to participants.

Moreover, the trial participant may be geographically distant from the investigator and/or the rest of the research team. Certain trial activities may occur remotely or may be performed by the trial participant’s individual HCP, local clinical staff, a sub-investigator, remote research staff, or a mobile HCP. Due to potential ambiguities in language between “routine care” and “practice of medicine” as opposed to “clinical trial-related activities,” the separation of routine care/practice of medicine and clinical trial activities should be well defined in the protocol to clarify the trial team’s roles and responsibilities, and should be in accordance with applicable FDA regulations (i.e., 21 CFR 11, 50, 54, 56, 312, and 812) and guidances [34–46].

In assessing the level of involvement of local providers, a key determination for clinical trials will be whether to identify HCPs on Form FDA 1572—whether HCPs assist the investigator by making direct and significant contribution to the data [34]. Relevant considerations for determining who and which facilities should be included on the Form FDA 1572 (“Statement of the Investigator”) [34] and delegation log include the investigator’s plan to supervise trial conduct, the credentials and licensure of the local HCP/sub-investigator/other trial staff, and the protocol’s language about trial-related procedures that can be performed by a local HCP and/or other trial personnel. In addition to Form 1572, the delegation log should include individual HCPs when they will provide specific services as part of the clinical trial.

### Safety Monitoring

Remote safety monitoring procedures should be well documented and investigative staff should be trained on processes that are unique to DCTs. For example, training may include ensuring that the trial participant at a remote location knows how to obtain information to address possible adverse events (e.g., a list of approved local healthcare facilities and/or clinicians for emergent issues related to the trial). Protocol-specific safety monitoring and communication escalation plans should be developed for trial participants, trial personnel, third-party vendors, and investigators. A potential safety issue’s effect on the use of mobile/remote technologies by the participant to report an adverse event (e.g., blurred vision may make it difficult to use a tablet, phone app, or computer) may be an important consideration. Sites should be properly resourced to review data in a timely manner and have contacts/infrastructure in place to react over distance (with or without a local investigator) accordingly, as necessary.

DCTs that rely on individual participants as a partner in the research and safety reporting will require effective participant training and education. Designing and incorporating simple safety reporting mechanisms using mobile technologies should be considered and potentially require more active participant engagement and understanding of, as well as comfort with, these technologies. Explicit protocol inclusion/exclusion criteria may help to ensure participants have the requisite technological skill and means to be successful in these trials, while balancing justice and equity considerations for participant selection. Here, again, tailored engagement of a suitable target population may increase participant comfort and convenience, improving satisfaction with and reinforcing the ultimate benefits of DCTs.

## Discussion

As the costs of medical product development continue to increase, the industry must adopt new and more efficient approaches, such as DCTs. DCTs may be associated with additional expenses initially, yet once established, they hold promise to be more cost- and time-efficient and to provide quality data. Embracing decentralized methodologies while engaging in early and ongoing dialogue with the FDA on conduct and design will guide initial DCTs toward success, which in turn will provide experiential evidence to drive future guidance on DCTs for the industry.

### Suitability

Not all clinical trials are suitable for decentralization. Where infrastructure is already established for an ongoing trial, amending the protocol to introduce remote or decentralized methodologies may be ideal incremental step. Still, several factors affect the evaluation of whether a proposed trial is suitable for a DCT or hybrid DCT approach. A critical consideration is an understanding of the safety profile of the IMP. A DCT strategy would probably not be suitable for a new molecular entity where the safety profile is unknown, but could be appropriate for trials involving an IMP with a well-known safety profile, perhaps being studied for a new indication. Considerations should be given regarding whether there is sufficient information about the IMP available to warrant continued developmental work outside of the brick and mortar trial setting, and whether the participant population is appropriate for a DCT. The latter should be a determination made by evaluating several factors, including the setting where the population traditionally receives care and whether the population is stable enough for participation in a trial with remote components.

Mobile technology offers new ways to capture objective measurements as clinical trial participants go about their daily lives by utilizing novel endpoints. These novel endpoints have the potential to provide high-quality data pertaining to outcomes that are meaningful to patients and enable decentralization of trials. There is, however, a need to consider how novel study endpoints will be measured and whether additional validation efforts are required to ensure that the decentralized and traditional measurements are comparable. Depending upon suitability, the use of mobile technologies in fit-for-purpose protocol designs can capture the same clinical endpoints using different technology or potentially augment or replace traditional validated assessments. For example, a wearable with an electrocardiogram might be appropriate for selected DCTs to monitor heart rate and rhythm. Continuous monitoring may result in far more data being transmitted than the snapshot of data collected during research visits in traditional trials. Such devices must be validated in order to ensure they reliably provide the necessary safety data, accounting not only for the means of data collection but also for the analysis of the data collected [32].Such devices must be validated in order to ensure they reliably provide the necessary safety data, accounting not only for the means of data collection but also for the analysis of the data collected [42].

### Participant Centricity

The aspiration of tailoring clinical research to individual participants to create net efficiencies may seem incompatible with the elimination of in-person visits with a single investigator at a local investigative site in a nearby central location. To overcome this perception, DCT sites must have the support and demonstrate the will to focus on the individual research participant while implementing clinical research from a distant location, potentially across multiple legal jurisdictions, and with different communities of interest. The patient perspective in the trial design and conduct is critical.

For decades, clinical trials have been conducted across states and even countries, and trial staff have managed the complexity that this necessarily entails. However, the day-to-day operations and authority have typically been vested in many local investigators and sites. DCTs, while decentralized from the perspective of moving research away from traditional sites and into the home of each participant, are at the same time increasingly centralized by requiring fewer investigative sites. Additionally, such a paradigm shift may challenge appropriate consideration of local norms, a hallmark of IRB review. In order to empower the individual participant, sites with larger participant pools will likely require partnerships with, for example, local and mobile HCPs, to account for additional participants under their care and manage any distance involved that cannot be bridged by telemedicine.

## Conclusions

Use of remote or decentralized methodologies to conduct clinical trials has the potential to improve the efficiency of medical product development. With this goal in mind, CTTI developed recommendations to address the most prevalent legal, regulatory, and practical issues regarding DCTs, including aspects related to trial design and conduct, telemedicine state licensing, drug supply chain, mobile HCPs, investigator delegation and oversight, and safety monitoring. As in most fields, with the advent of new technologies (or older technologies applied in new ways), perception, practicable regulation, and responsible adoption struggle to keep pace. The CTTI DCT project team has identified perceived and actual barriers to decentralization and has developed recommendations that are intended to demonstrate the merits and drive broader use of DCTs.
